# ATP/IL-33-Co-Sensing by Mast Cells (MCs) Requires Activated c-Kit to Ensure Effective Cytokine Responses

**DOI:** 10.3390/cells12232696

**Published:** 2023-11-24

**Authors:** Johanna Seifert, Claudia Küchler, Sebastian Drube

**Affiliations:** Institut für Immunologie, Universitätsklinikum Jena, Friedrich-Schiller-Universität Jena, Leutragraben 3, 07743 Jena, Germany

**Keywords:** mast cells, ATP/IL-33 co-sensing, stem cell factor (SCF), p65/RelA, NFAT

## Abstract

Mast cells (MCs) are sentinel cells which represent an important part of the first line of defense of the immune system. MCs highly express receptors for danger-associated molecular patterns (DAMPs) such as the IL-33R and P2X7, making MCs to potentially effective sensors for IL-33 and adenosine-triphosphate (ATP), two alarmins which are released upon necrosis-induced cell damage in peripheral tissues. Besides receptors for alarmins, MCs also express the stem cell factor (SCF) receptor c-Kit, which typically mediates MC differentiation, proliferation and survival. By using bone marrow-derived MCs (BMMCs), ELISA and flow cytometry experiments, as well as p65/RelA and NFAT reporter MCs, we aimed to investigate the influence of SCF on alarmin-induced signaling pathways and the resulting cytokine production and degranulation. We found that the presence of SCF boosted the cytokine production but not degranulation in MCs which simultaneously sense ATP and IL-33 (ATP/IL-33 co-sensing). Therefore, we conclude that SCF maintains the functionality of MCs in peripheral tissues to ensure appropriate MC reactions upon cell damage, induced by pathogens or allergens.

## 1. Introduction

One part of the innate immune system are MCs, which facilitate the defense against pathogens by releasing proteases, anti-microbial peptides and histamines [1]. MC activation also results in the production of cytokines and chemokines, which mediate the attraction of neutrophils to the side of infection [2,3,4]. The differentiation, survival and proliferation of MCs is mediated by the stem cell factor (SCF)-induced c-Kit activation, which ensures MC homeostasis in peripheral tissues [5,6,7,8]. Thereby, the SCF-induced activation of STAT3/5 [7,9] mediates MC differentiation, whereas MC survival and proliferation are rather mediated by the PI3K-PKB/Akt module and p38 [10,11,12]. In peripheral tissues, mature MCs are typically activated by IgE molecules which bind to the FcεRI on MCs [13]. The binding of antigens to FcεRI-bound IgE and the resulting FcεRI crosslinking results in fast MC responses, characterized by the production of cytokines, degranulation and thus, the release of granule-stored proteases and/or histamines [13]. Besides the FcεRI receptor, MCs also highly express the IL-33R to sense IL-33 and P2X7 to sense ATP [14,15]. IL-33 is predominantly expressed as a nuclear cytokine in cells with barrier functions such as endothelial and epithelial cells, as well as in fibroblasts, and it regulates the expression of different genes in these cells [16,17]. In contrast to IL-33, ATP is ubiquitously produced during glycolysis and the citrate cycle. Of note, both IL-33 and ATP are released upon damage to the integrity of cellular barriers [18]. However, despite the location of MCs in peripheral tissues and the high expression of IL-33R and P2X7 in MCs, IL-33 or ATP alone are only mild and limited MC activators [14]. In fact, the co-sensing of ATP and IL-33 by MCs is required to induce the strong production of a broad spectrum of pro-inflammatory cytokines and degranulation [2,14]. This ATP/IL-33-induced hyperactivated MC phenotype mediates the vascular response, culminating in contact hypersensitivity reactions (CHSs) independent from the adaptive immune system [2,14]. In addition to such allergic reactions, we suggest that the co-sensing of ATP and IL-33 by MCs also contributes to the development of pulmonary diseases such as COVID-19. Interestingly, in lung tissues of SARS-CoV2-infected humans and mice, hyperactivated MCs were found [19,20,21]. Thereby, the presence of hyperactivated MCs correlated with the strongly elevated levels of IL-33 [22,23], ATP [24] and MC proteases [19] in the serum of COVID-19 patients. Therefore, ATP/IL-33 co-sensing in infected lung tissues might be a potential mechanism explaining the hyperactivated MC phenotype in SARS-CoV2-infected individuals [14]. Confirming this, the role of activated MCs and of IL-33 in COVID-19 pathogenesis has been shown recently [21,25], demonstrating that released alarmins and MCs are indeed important for hyperinflammatory reactions in peripheral tissues. In this work, we show that in addition to ATP/IL-33 co-sensing, SCF is a further stimulus which enhances the hyperinflammatory MC phenotype. Therefore, targeting the SCF/c-Kit axis and/or ATP/IL-33 co-sensing might be beneficial to treat MC-mediated hyperinflammatory responses.

## 2. Materials and Methods

### 2.1. Mice

We used sex- (either all male or all female mice) and age-matched (8–12 weeks) wt C57/B6 mice, which were maintained under SPF conditions at the Animal Research Facility of the Jena University Hospital. The organ isolation was approved by the Thüringer Landesamt für Lebensmittelsicherheit und Verbraucherschutz (TLLV); Bad Langensalza; the license for the Institute of Immunology, Jena was twz-36-2017.

### 2.2. Bone Marrow-Derived Mast Cell (BMMC) Generation and Culture

The isolated bone marrow cells were cultured in IMDM (PAA) supplemented with 10% FCS, 100 U/mL penicillin, 100 mg/mL streptomycin, 50 mM 2-mercaptoethanol and 20 ng/mL IL-3 (conditioned medium from 20 ng/mL X63Ag-653 BPV-rmIL-3 supernatant, the source of IL-3). The removal of adherent cells from the cultures was achieved by refreshing the media every second day during the 4 weeks of culture. After 4 weeks, cultures were tested for the content of FcεRI+/c-Kit+ cells (BMMCs). When the content of FcεRI+/c-Kit+ cells reached 95%, the cells were used for an additional 4–5 weeks. To maintain the number of BMMCs, the medium was refreshed twice a week until the 9th week.

### 2.3. Culture of p65/RelA-EGFP and NFAT-EGFP Reporter MC/9 Cells

p65/RelA-EGFP and NFAT-EGFP reporter MC/9 cells were kindly provided by Dr. Zhang [26]. These cells were cultured in RPMI (Sigma-Aldrich, Taufkirchen, Germany) (supplemented with 10% FCS, 100 U/mL penicillin, 100 mg/mL streptomycin, 50 mM β-mercaptoethanol and 20 ng/mL IL-3 (conditioned medium from X63Ag-653 BPV-rmIL-3 cells)).

### 2.4. Stimulation of BMMC

BMMCs (10^6^ cells/mL) were starved for IL-3 (1 h). Afterwards, BMMCs were directly single-stimulated with SCF (50 ng/mL), ATP (in different concentrations) or IL-33 (in different concentrations). In some experiments, BMMCs were treated with a vehicle (DMSO (Sigma-Aldrich, Taufkirchen, Germany) as controls, Takinib [27] (10 µM), A438079 (50 µM) or Cyclosporine A (2 µg/mL) (all Selleckchem, Munich, Germany) for 30 min.

The concentrations of the inhibitors were determined regarding their potential to induce cell death. The inhibitor concentrations which do not induce cell death were used. DMSO- or inhibitor-treated BMMCs were then left unstimulated or were single-stimulated with ATP (as indicated in the figures) (Sigma-Aldrich, Taufkirchen, Germany) or IL-33 (as indicated in the figures) (Peprotech, Hamburg, Germany) or with ATP together with IL-33 (as indicated in the figures). In some experiments, prior to stimulation with ATP or IL-33 or both in combination (as indicated in the figures), BMMCs were pre-stimulated with SCF (50 ng/mL) (Peprotech, Hamburg, Germany) for 30 min. Supernatants were collected (after 24 h) and were analyzed for released cytokines by ELISA with matched paired antibodies.

### 2.5. BMMC Staining and Flow Cytometry

For the quality control of BMMC cultures, cells were harvested and were washed with PBA buffer (PBS supplemented with BSA (Roth, Karlsruhe, Germany)). To block unspecific bindings of the specific antibodies, BMMCs were treated with rat-IgG (Jackson, Baltimore, MD, USA). BMMCs were afterwards stained with PE-conjugated anti-c-Kit and FITC-conjugated anti-FcεRI (all BioLegend, San Diego, CA, USA) and were then treated with Propidium iodide (PI) for dead cell exclusion during measurement. Samples were recorded by using the LSR II flow cytometer (BD). To investigate MC degranulation, BMMCs were seeded (1 × 10^6^ cells/mL) in IL-3-free medium. After 1 h, BMMCs were pre-stimulated with SCF (50 ng/mL) (Peprotech, Hamburg, Germany) for 30 min. Afterwards, BMMCs were stimulated with ATP (250 µM) (Sigma-Aldrich, Taufkirchen, Germany) or IL-33 (25 ng/mL) (Peprotech, Hamburg, Germany) or ATP together with IL-33 for 30 min. BMMCs were harvested, washed with PBA and treated with rat-IgG (Jackson, Baltimore, MD, USA) to block unspecific antibody binding. BMMCs were then stained with APC-conjugated anti-c-Kit and PE-conjugated anti-CD107α (both BioLegend, San Diego, CA, USA). After incubation (30 min), BMMCs were washed with PBA and were treated with PI-PBA buffer for dead cell exclusion. Samples were recorded by using the LSR II flow cytometer (BD). For the detection of cell death induction by the inhibitors, BMMCs were seeded in IL-3-free medium (1 × 10^6^ cells/mL). After 1 h in culture, BMMCs were treated with vehicle (DMSO) or inhibitors (as indicated in the figures). Afterwards, cells were harvested, centrifuged and washed with PBA buffer. BMMCs were then treated with PI-PBA buffer to determine the percentages of dead cells. Samples were recorded by using the LSR II flow cytometer (BD). All measurements were analyzed for the expression of EGFP by FlowJo 9 (Treestar Inc., Ashland, OR, USA).

### 2.6. Detection of EGFP in p65/RelA-EGFP- or NFAT-EGFP-MC/9 Mast Cells

p65/RelA-EGFP- or NFAT-EGFP-MC/9 cells were seeded (10^6^ cells/mL) in IL-3-free RPMI medium (supplemented with 10% FCS, 100 U/mL penicillin, 100 mg/mL streptomycin, 50 mM 2-mercaptoethanol). After 1 h, cells were pre-stimulated with SCF (50 ng/mL) (in some experiments) for 30 min. BMMCs were then either left unstimulated or were stimulated with ATP (250 µM) or IL-33 (25 ng/mL) or both together. For the detection of produced EGFP in stimulated p65/RelA-EGFP- or NFAT-EGFP-MC/9 MCs, cells were harvested (after 24 h) and were washed with PBA. Cells were collected and treated with PI-PBA buffer for dead cell exclusion. Samples were recorded by using the LSR II flow cytometer (BD) and were then analyzed for the expression of EGFP by FlowJo 9 (Treestar Inc., Ashland, OR, USA).

### 2.7. Statistical Analysis

All experiments were performed at least 3 times (if not otherwise stated). For ELISA and flow cytometry experiments, we show the summary of results obtained from at least *n* = 3 biological replicates of wt BMMCs. One biological BMMC replicate consists of pooled bone marrow cells from *n* = 2 mice. For the quantification of flow cytometry experiments, the summary of at least *n* = 3 independent experiments (if not otherwise shown) is shown. Shown is the mean ± SEM. For statistical analysis, we used SigmaPlot 13.0 (Systat Software, Inc., Düsseldorf, Germany) and calculated the statistical significance by using the Student’s *t*-test. Significance was accepted for *p* ≤ 0.05 (* *p* ≤ 0.05; ** *p* ≤ 0.01; *** *p* ≤ 0.001; ns: not significant).

## 3. Results

### 3.1. ATP/IL-33-Co-Sensing by MCs

Recently, we found that the co-sensing of ATP and IL-33 synergistically enhanced the cytokine production in MCs [14]. Here, we determined the concentrations of ATP and IL-33, which are sufficient to induce enhanced cytokine production in MCs. As shown in Figure S1a–f, IL-33 and ATP in different concentrations alone induced a mild production of the pro-inflammatory cytokines IL-2, IL-5, IL-6, IL-13 and GM-CSF. Thereby, the combination of ATP in a concentration of 250 µM with IL-33 is sufficient to boost the induced cytokine production compared to either stimulus alone (Figure S1a–f). These data confirmed our recent data [14] but furthermore show that lower ATP and IL-33 concentrations are sufficient to synergistically enhance the induced cytokine responses in MCs compared to either stimulus alone. Due to these results, we used ATP in a concentration of 250 µM.

### 3.2. SCF Supports the Cytokine Production Induced by ATP/IL-33 Co-Sensing

**Figure 1:**
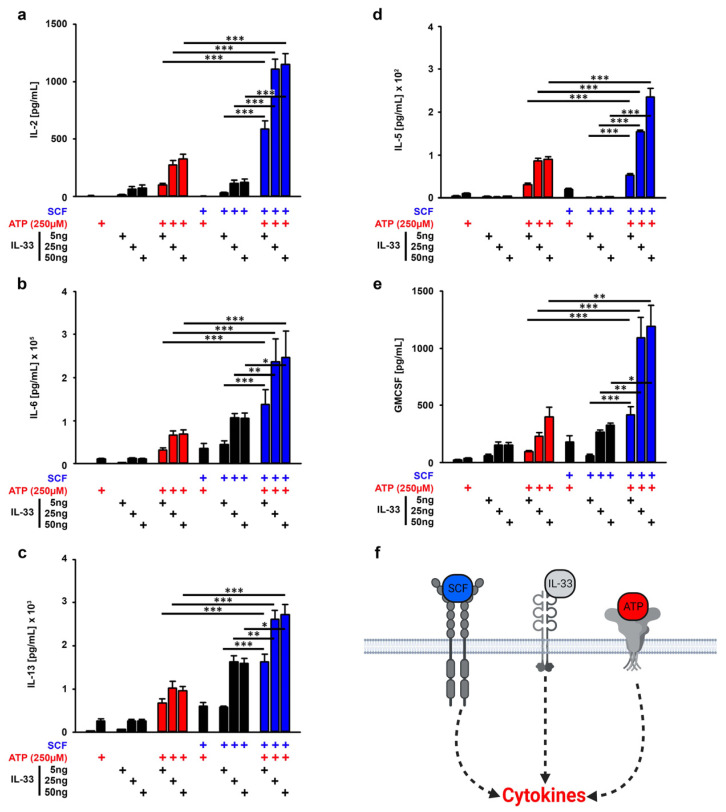
SCF amplifies the ATP/IL-33-induced cytokine response. (**a**–**e**) BMMCs cells were either single-stimulated with ATP (250 µM) or IL-33 (as indicated), co-stimulated with ATP/IL-33, SCF/ATP or SCF/IL-33 or were triple-stimulated with SCF/ATP/IL-33. In samples with SCF (50 ng/mL), cells were pre-stimulated with SCF for 30 min prior to the stimulation with the alarmins ATP and/or IL-33. After 24 h, supernatants were collected and analyzed for IL-2 (**a**), IL-6 (**b**), IL-13 (**c**), IL-5 (**d**) or GM-CSF (**e**). (**f**) Shown is a cartoon of the triple stimulation of BMMCs with SCF, ATP and IL-33 (created with Biorender). Shown is the SEM of *n* = 6 biological replicates for all cytokines (* *p* ≤ 0.05; ** *p* ≤ 0.01; *** *p* ≤ 0.001). + indicates the presence of ATP, + indicates the presence of IL-33, + indicates the presence of SCF.

ATP and IL-33 synergistically enhanced the cytokine production in MCs in vitro (Figure S1a–f and [14]). In line with these findings, the 2,4-dinitrofluorobenzene (DNFB)-induced contact allergy depends on IL-33 and ATP in vivo [2]. Given that SCF is needed to ensure effective MC effector functions [28,29], we examined whether the presence of SCF also supports the cytokine production resulting from ATP/IL-33 co-sensing. SCF alone only induced a faint production of IL-6 and IL-13 (Figure S2a–f). In contrast to this, ATP alone induced a strong production of IL-5 and IL-6, whereas IL-33 alone induced a strong production of IL-2, IL-6, IL-13 and GMCSF but not of IL-5 (Figure S2a–f). Interestingly, despite the missing effective cytokine production, SCF enhanced all alarmin-induced cytokine responses (Figure 1a–f). However, the most efficient cytokine production was induced by SCF combined with ATP/IL-33 if compared to the stimulations with ATP/IL-33, SCF/ATP and SCF/IL-33 (Figure 1a–f, blue bars). Thereby, IL-33 in a concentration of 25 ng/mL is sufficient to induce these effects. Therefore, we used this IL-33 concentration for the further experiments.

### 3.3. The SCF and/or IL-33 Did Not Influence the ATP-Induced MC Degranulation

**Figure 2:**
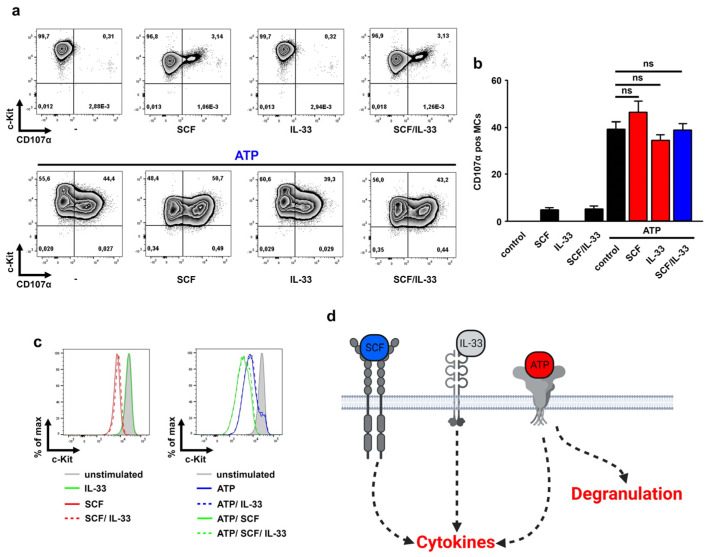
SCF did not influence MC degranulation. (**a**–**c**) BMMCs cells were either single-stimulated with SCF (50 ng/mL), IL-33 (25 ng/mL) or ATP (250 µM); co-stimulated with SCF/IL-33, SCF/ATP or ATP/IL-33; or were triple-stimulated with SCF/ATP/IL-33. Thereby, in samples with SCF (50 ng/mL), cells were pre-stimulated with SCF for 30 min prior to the stimulation with the alarmins ATP and/or IL-33. After 30 min, cells were harvested and analyzed by flow cytometry. (**a**) Shown is the c-Kit/CD107α staining. (**b**) Shown is the SEM of *n* = 3 biological replicates for all stimulations (ns: not significant). (**c**) Shown is the c-Kit staining. (**d**) Shown is a cartoon of the triple stimulation of BMMCs with SCF, ATP and IL-33 and the resulting cytokine production and MC degranulation (created with Biorender https://www.biorender.com/).

ATP induces MC degranulation which is not influenced by IL-33 [14]. Given that SCF enhances the cytokine responses induced by ATP/IL-33 co-sensing, we speculated that SCF also supports the ATP-induced MC degranulation. To investigate MC degranulation, we used CD107α [30] which is only detectable on degranulated MCs [14]. As shown in Figure 2a,b, neither SCF or IL-33 alone, nor the combination of both, effectively induced MC degranulation. In contrast to this, stimulation with ATP strongly induced the surface expression of CD107α which demonstrates MC degranulation (Figure 2a,b). However, neither SCF nor IL-33 or both together (SCF/IL-33) affected the ATP-induced CD107α surface expression (Figure 2a,b). In contrast to this, the ATP-induced internalization of c-Kit [14] was further enhanced in the presence of SCF (Figure 2c). This demonstrated that the ATP/SCF stimulation converts c-Kit^high^/CD107α^neg^ MCs into c-Kit^low^/CD107α^high^ MCs (Figure 2c). These data show that SCF supported the ATP/IL-33-induced cytokine response but did not influence MC degranulation (Figure 2d).

### 3.4. TAK1 Is Essential for the SCF-Supported ATP/IL-33 Co-Sensing

**Figure 3:**
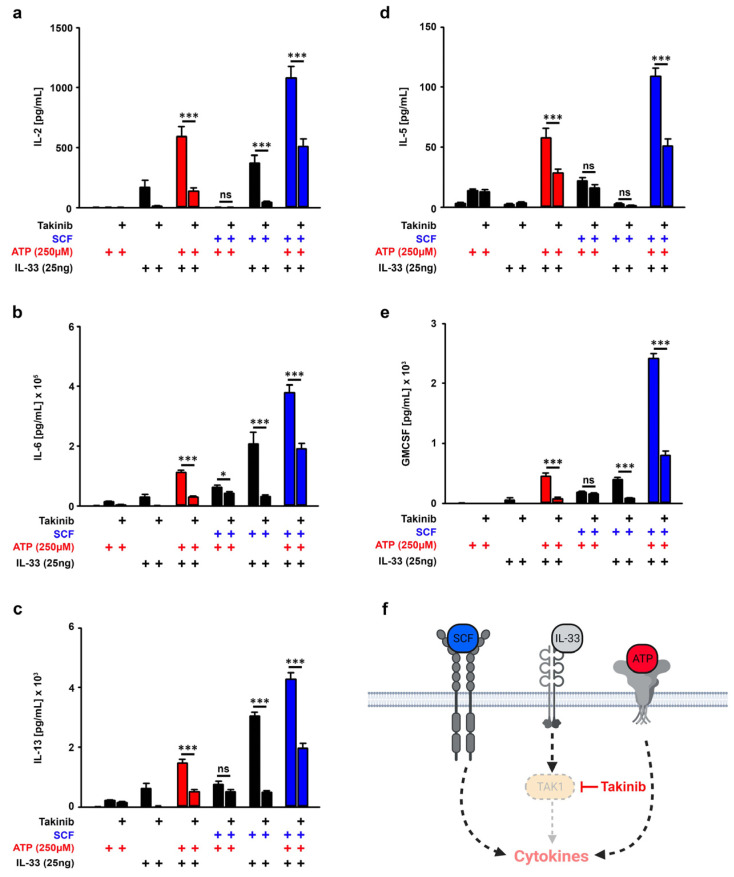
TAK1 amplifies the SCF/ATP/IL-33-induced cytokine response. (**a–e**) BMMCs were pre-incubated with Takinib (10 µM) for 30 min (as indicated). Afterwards, BMMCs were either single-stimulated with ATP (250 µM) or IL-33 (25 ng/mL), co-stimulated with ATP/IL-33, SCF/ATP or SCF/IL-33 or were triple-stimulated with SCF/ATP/IL-33. In samples with SCF (50 ng/mL), cells were pre-stimulated with SCF for 30 min prior to the stimulation with the alarmins ATP and/or IL-33. After 24 h, supernatants were collected and analyzed for IL-2 (**a**), IL-6 (**b**), IL-13 (**c**), IL-5 (**d**) or GM-CSF (**e**). (**f**) Shown is a cartoon of the triple stimulation of BMMCs with SCF, ATP and IL-33 in the presence of Takinib (created with Biorender). (**a**–**e**) Shown is the SEM of *n* = 5 biological replicates for IL-2, IL-5 and IL-6 and *n* = 3 biological replicates for IL-13 and GM-CSF (* *p* ≤ 0.05; *** *p* ≤ 0.001; ns: not significant). + indicates the presence of ATP, + indicates the presence of IL-33 or Takinib, + indicates the presence of SCF.

SCF only supported the ATP/IL-33-induced cytokine productions but not the degranulation. Therefore, we focused on the investigation of the mechanism behind the cytokine production in response to SCF/ATP/IL-33 stimulations. First, we determined the role of the IL-33-induced signaling cascade. IL-33 (but neither ATP nor SCF) induces TAK1-dependent signaling pathways [14]. Thereby, TAK1 is an important signaling hub downstream of the IL-33R/MyD88 module and upstream of the IκB-kinases IKK1/2. Thus, the inhibition of TAK1 blocks the activation of p65/RelA and the activation of MAP-kinases. To specifically block IL-33-induced signaling pathways, we used the highly specific pharmacological TAK1 inhibitor Takinib [27] which did not induce cell death in BMMCs (Figure S3). Treatment with Takinib completely blocked the cytokine production induced by single stimulation with IL-33. However, when IL-33 was combined with ATP or SCF as well as with ATP/SCF, Takinib did not completely block, but only reduced the resulting cytokine production (Figure 3a–f). This indicated that either the Takinib concentration was not sufficient, or that co- or triple stimulations activate additional TAK1-independent signaling pathways. Nevertheless, the fact that Takinib reduced the cytokine responses in co- and triple-stimulated MCs demonstrated that TAK1 is an essential component involved in the cytokine productions induced by the co-sensing of ATP and IL-33 in the presence of SCF.

### 3.5. P2X7 Is Essential for the SCF-Supported ATP/IL-33 Co-Sensing

**Figure 4:**
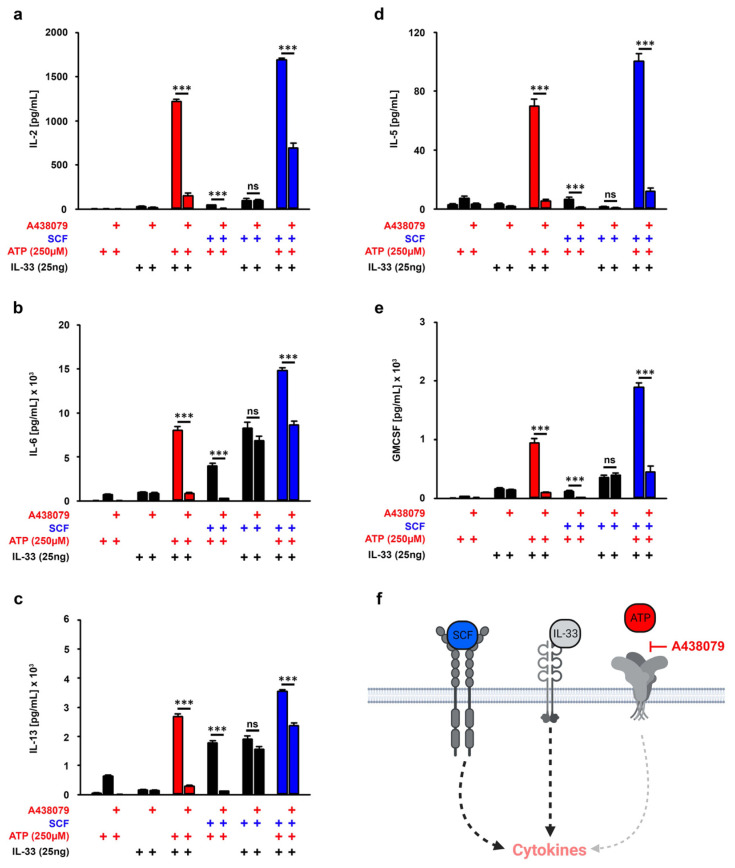
P2X7 amplifies the SCF/ATP/IL-33-induced cytokine response. (**a**–**e**) BMMCs were pre-incubated with A438079 (50 µM) for 30 min (as indicated). Afterwards, BMMCs were either single-stimulated with ATP (250 µM) or IL-33 (25 ng/mL), co-stimulated with ATP/IL-33 or SCF/ATP SCF/IL-33 or were triple-stimulated with SCF/ATP/IL-33. In samples with SCF (50 ng/mL), cells were pre-stimulated with SCF for 30 min prior to the stimulation with the alarmins ATP and/or IL-33. After 24 h, supernatants were collected and analyzed for IL-2 (**a**), IL-6 (**b**), IL-13 (**c**), IL-5 (**d**) or GM-CSF (**e**). (**f**) Shown is a cartoon of the triple stimulation of BMMCs with SCF, ATP and IL-33 in the presence of A438079 (created with Biorender). (**a**–**e**) Shown is the SEM of *n* = 5 biological replicates for GM-CSF and IL-5 and IL-6 and *n* = 3 biological replicates for IL-2, IL-6 and IL-13 (*** *p* ≤ 0.001; ns: not significant). + indicates the presence of ATP or A438079, + indicates the presence of IL-33, + indicates the presence of SCF.

ATP binds and activates the purinergic receptor P2X7. However, ATP is cleaved into ADP and AMP by CD39 on MCs [15,31,32]. Given that ADP and AMP act via purinergic P2Y and adenosine receptors which are also expressed on MCs [14], the effects of SCF on the cytokine responses might be mediated by ATP degradation products. If P2X7 is the only receptor which mediates the increased cytokine productions induced by SCF/ATP/IL-33, the blocking of P2X7 should reduce the cytokine productions to the level which was induced by SCF/IL-33 co-stimulation. As an antagonist for ATP binding to P2X7, we used the ATP antagonist A438079, which did not show any cytotoxic effects in BMMCs (Figure S3). As shown in Figure 4a–f, A438079 blocked the cytokine production induced by single stimulation with ATP and induced by co-stimulation with ATP/IL-33 and ATP/SCF, demonstrating that A438079 selectively blocked the ATP-induced signaling (Figure 4a–f). However, except from the IL-2 production, A438079 reduced the SCF/ATP/IL-33-induced production of IL-6, IL-13, IL-5 and GMCSF to levels which were induced by SCF/IL-33 (Figure 4a–f, blue bars). This indicated that only the increased IL-2 production in response to SCF/ATP/IL-33 is not exclusively mediated by P2X7. These data show that the SCF-supported ATP/IL-33 cytokine responses are predominantly mediated by P2X7 and not by ATP degradation products.

### 3.6. CaN Downstream of P2X7 Is Essential for the SCF-Supported ATP/IL-33 Co-Sensing

**Figure 5:**
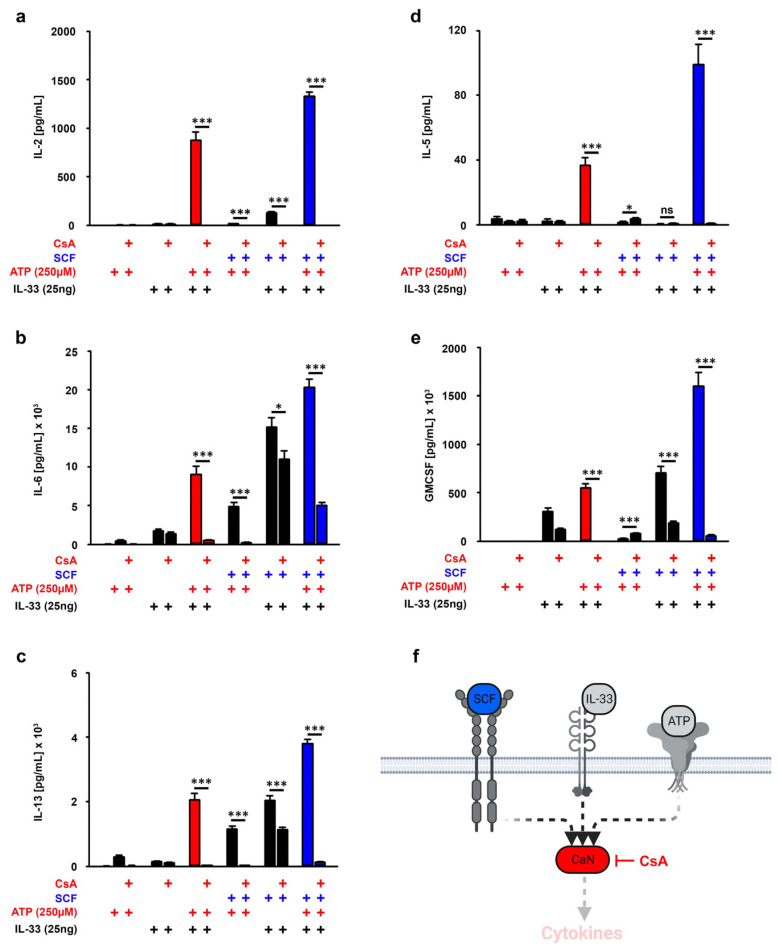
CaN amplifies the SCF/ATP/IL-33-induced cytokine response. (**a**–**e**) BMMCs were pre-incubated with CaN (2 µg/mL) for 30 min (as indicated). Afterwards, BMMCs were either single-stimulated with ATP (250 µM) or IL-33 (25 ng/mL), co-stimulated with ATP/IL-33 or SCF/ATP SCF/IL-33 or were triple-stimulated with SCF/ATP/IL-33. In samples with SCF (50 ng/mL), cells were pre-stimulated with SCF (in blue) for 30 min prior to the stimulation with the alarmins ATP and/or IL-33. After 24 h, supernatants were collected and analyzed for IL-2 (**a**), IL-6 (**b**), IL-13 (**c**), IL-5 (**d**) or GM-CSF (**e**). (**f**) Shown is a cartoon of the triple stimulation of BMMCs with SCF, ATP and IL-33 in the presence of CaN (created with Biorender). (**a**–**e**) Shown is the SEM of *n* = 3 biological replicates for all cytokines. (* *p* ≤ 0.05; *** *p* ≤ 0.001; ns: not significant). + indicates the presence of ATP or CsA, + indicates the presence of IL-33, + indicates the presence of SCF.

Next, we wanted to investigate the role of the phosphatase Calcinuerine (CaN), the most important signaling molecule downstream of P2X7 [33]. To block CaN, we used the highly specific CaN inhibitor Cyclosprine A (CsA) which is not cytotoxic in BMMCs (Figure S3). While CsA did not affect the cytokine response induced by single stimulation with IL-33, CsA blocked the cytokine responses induced by ATP alone and induced by ATP/IL-33 and SCF/ATP (Figure 5a–f). Unexpectedly, CsA also reduced the production of IL-2, IL-6, IL-13 and GM-CSF which were induced by SCF/IL-33 co-stimulation (Figure 5a–f). This shows that CaN is not only activated by ATP but also by co-stimulation with SCF and IL-33. Thereby, neither SCF nor IL-33 alone induced CsA-sensitive effector functions. Next, we determined the role of CaN in MCs triple-stimulated with SCF, ATP and IL-33.

As shown in Figure 5a–f (blue bars), CsA treatment resulted in a complete inhibition of the cytokine responses induced by ATP/IL-33 co-sensing in the presence of SCF. These data show that CaN is an essential signaling component in MC co-sensing ATP/IL-33 in the presence of SCF.

### 3.7. SCF Predominantly Enhances the p65/RelA Activation Induced by ATP/IL-33 Co-Sensing

**Figure 6:**
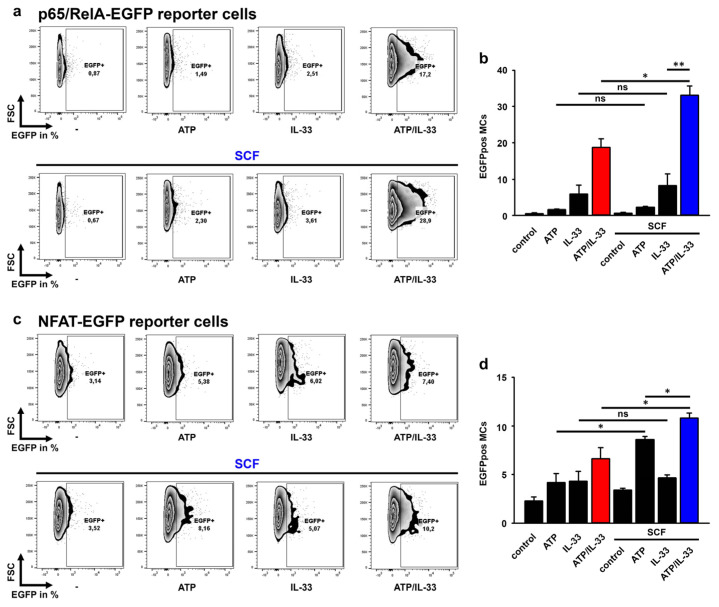
The activation of p65/RelA and NFAT is effectively activated in MC/9 cells stimulated with SCF/ATP/IL-33. (**a**–**d**) p65/RelA-EGFP-MC/9 (**a**,**b**) or NFAT-EGFP-MC/9 cells (**c**,**d**) were single-stimulated with ATP (250 µM) or IL-33 (25 ng/mL) or co-stimulated with ATP/IL-33. In some samples, SCF (50 ng/mL) was pre-stimulated prior to stimulation with ATP, IL-33 or ATP/IL-33. After 24 h, cells were harvested and analyzed by flow cytometry. Shown is one representative experiment out of three. (**b**,**d**) Shown is the SEM of *n* = 3 independent experiments with p65/RelA-EGFP-MC/9 (**b**) and NFAT-EGFP-MC/9 (**d**) (* *p* ≤ 0.05; ** *p* ≤ 0.01; ns: not significant).

Next, we aimed to determine the influence of SCF on the ATP/IL-33-induced activation of p65/RelA and NFAT by using MC/9 MCs containing a p65/RelA-EGFP or a NFAT-EGFP reporter construct. In contrast to IL-33 alone, ATP and SCF failed to induce the effective activation of p65/RelA (Figure 6a,b). However, ATP increased the IL-33-induced p65/RelA activation (Figure 6a,b; red bar in Figure 6b). While SCF did not significantly influence the ATP- and IL-33-induced p65/RelA activation, it strongly enhanced the p65/RelA activation induced by the co-sensing of ATP and IL-33 (Figure 6a, blue bar in Figure 6b). Next, we tested the influence of SCF on the NFAT activation. Interestingly, compared to stimulation with ATP or IL-33 alone, ATP/IL-33 co-sensing is required to effectively induce the activation of NFAT (Figure 6c,d; red bar in Figure 6d). Furthermore, whereas SCF alone did not activate NFAT, it was required to enable ATP or the combination of ATP and IL-33 to induce the effective activation of NFAT (Figure 6c,d; blue bar in Figure 6d). Thereby, the co-sensing of ATP and IL-33 in combination with SCF induced the most effective NFAT activation in MCs (Figure 6c,d; blue bar in Figure 6d). Together, these data show that SCF increased the p65/RelA and NFAT activation induced by the co-sensing of ATP and IL-33.

## 4. Discussion

**Figure 7:**
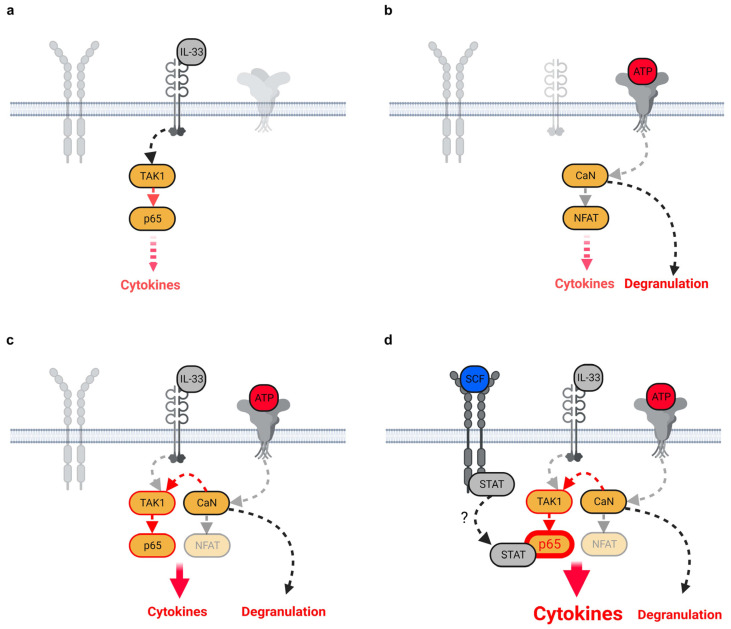
SCF supports the cytokine production but not degranulation induced by ATP/IL-33 co-sensing. (**a**) The IL-33-induced cytokine production [40]. IL-33 induces a TAK1-dependent activation of p65/RelA which results in the production of IL-2, IL-6 and GMCSF. Thereby, IL-33 is not able to induce MC degranulation. (**b**) The ATP-induced cytokine production [14]. ATP induces a Ca^2+^-dependent activation of the CaN-NFAT module. This results in the production of IL-5, IL-6 and IL-13 and MC degranulation. (**c**) Co-sensing of ATP and IL-33 [14]. IL-33 activates the TAK1-p65/RelA signaling pathway. Activated TAK1 is then targeted by ATP-activated CaN which prolongs the TAK1 activity and thus the activity of p65/RelA [14]. The combination of enhanced p65/RelA activity and the activity of NFAT potentiated the production of IL-2, IL-5, IL-6, IL-13 and GMCSF [14]. Thereby, the ATP-induced MC degranulation remained unchanged [14]. (**d**) Co-sensing of ATP and IL-33 in the presence of SCF. IL-33 activates the TAK1-p65/RelA signaling pathway. Activated TAK1 is then targeted by ATP-activated CaN, which prolongs the TAK1 activity and the activity of p65/RelA [14]. The additional presence of SCF activates STATs. We speculate that activated STATs interact with and further enhance the activity of p65/RelA which is induced by ATP/IL-33 co-sensing. Thereby, the ATP-induced MC degranulation remains unchanged. (Figures were created with Biorender).

MCs are sentinel cells of the innate immune system in peripheral tissues and are thus part of the first line of defense [1]. To sense tissue injuries, induced by pathogens or allergens, MCs express several receptors for the detection of alarmins, among them being IL-33R to detect IL-33 and P2X7 to detect ATP [14]. Despite the high expression of both receptors, IL-33 and ATP alone are only weak activators of MCs, resulting in the limited production of cytokines [14] (Figure 7a,b). Interestingly, both alarmins in combination are required to induce a hyperactivated MC phenotype, which is characterized by the strong production of a broad spectrum of pro-inflammatory cytokines and by degranulation (Figure 7c; [14]). This combinatory ATP and IL-33 sensing (ATP/IL-33 co-sensing) is required to induce a vascular response culminating in allergic contact hypersensitivity reactions (CHSs) in mice [2]. We speculate that ATP/IL-33 co-sensing by MCs is not limited to allergies but also plays an important role in infectious diseases such as COVID-19, which is characterized by a massive destruction of lung tissues. Therefore, we think that the presence of hyperactivated MCs in lung tissues of SARS-CoV2-infected humans and mice might be mediated by released ATP and IL-33 [19,20,21]. Interestingly, the MC responsiveness in vitro and in vivo is essentially maintained by SCF and the resulting c-Kit-mediated signaling [5,6,7,8,34]. Thereby, activated c-Kit ensures that IL-33 can mediate a strong cytokine production in MCs in vitro. This phenomenon was also observed in a murine colon cancer model [34]. In this model, the combined presence of SCF and IL-33 is required for the accumulation of connective tissue MCs which secrete IL-6 and TNF-α and thus maintain the pro-inflammatory microenvironment. These data indicated that SCF might also be an essential component which maintains the functionality of MCs in response to ATP/IL-33 co-sensing. Indeed, the presence of SCF is a further essential stimulus, which supports the hyperinflammatory MC phenotype induced by ATP/IL-33 co-sensing. Thereby, SCF converts CaN-independent into CaN-dependent cytokine responses. This shows that CaN becomes a central signaling hub which completely mediates the production of cytokines when MCs are stimulated with SCF together with ATP and IL-33. However, the presence of SCF did not influence MC degranulation, demonstrating that SCF selectively supports the cytokine production induced by ATP/IL-33 co-sensing. The discrepancy between the enhanced cytokine production and the unchanged degranulation in SCF/ATP/IL-33-stimulated MC excludes the fact that SCF additionally enhances the ATP-induced upregulation of intracellular Ca^2+^ levels and thus the resulting CaN activity, since this would also increase the MC degranulation. Recently, we showed that the Ca^2+^-CaN module prolongs the IL-33-induced activation of the TAK1-p65/RelA signaling pathway (Figure 7c; [14]). We rather speculate that additional SCF-activated STATs cooperatively act together with ATP/IL-33-activated p65/RelA (Figure 7d; [33]). Thereby, STATs interact with and thus enhance the activity of p65/RelA, resulting in an increased cytokine production (Figure 7d). In this model, SCF selectively enhances the ATP/IL-33-induced cytokine production, whereas the induced degranulation remains unchanged (Figure 7d). In summary, our data indicate that the activation of p65/RelA, NFAT and STATs in response to the combinatory SCF, IL-33 and ATP stimulation mimics an antigen-dependent activation of MCs, which is normally induced by the IgE/FcεRI axis and depends on the same transcription factors. This culminates in a strong production of pro-inflammatory cytokines and in degranulation [9,35,36,37,38]. However, our data are based on experiments with soluble SCF (sSCF). Under physiological conditions, SCF is predominantly expressed as a membrane-bound form (mem-SCF) [39] which ensures final differentiation and survival of MCs in peripheral tissues. In comparison to sSCF, mem-SCF mediates a tonic, permanent c-Kit signal. We mimicked this permanent signal by pre-stimulating MCs with sSCF for 30 min prior to stimulation with IL-33 and/or ATP. Therefore, we speculate that similarly to sSCF, mem-SCF is also able to mediate effective alarmin-induced MC effector functions. In summary, our data show that also in the absence of the adaptive immune response, MCs can be strongly activated by alarmins such as ATP together with IL-33 and in the presence of SCF. This activation is thereby characterized by degranulation [14] and strong cytokine production. Of note, given that this mode of activation resembles the antigen/IgE/FcεRI-dependent activation of MCs, we hypothesize that the damage of the cellular integrity by pathogens or allergens in peripheral tissues results in strong MC activation by alarmins in combination with growth factors, such as SCF. However, this also demonstrates that the induction of the alarmin-induced hyperactivation of MCs in peripheral tissues is strongly controlled. The simultaneous release and sensing of ATP in combination with IL-33 and SCF must be fulfilled to induce the hyperactivated MC phenotype independently from the FcεRI. Consequently, this mechanism ensures the reaction of MCs to pathogens or allergens at a very early time point of the immune response and thus bridges the time until the cells of the adaptive immune system are differentiated, attracted and activated. Our data suggest that these mechanisms are also relevant for the development of cytokine storm syndromes (as shown in COVID-19 patients) or allergic reactions. Therefore, the targeting of either the SCF/c-Kit axis or ATP as well as combinatory therapies targeting SCF, IL-33 and/or ATP might be beneficial to treat MC-mediated hyperinflammatory responses.

## 5. Conclusions

SCF is essential for MCs to effectively react to ATP/IL-33 co-sensing. Therefore, we speculate that this mechanism also maintains the function of MCs in peripheral tissues to ensure appropriate MC effector functions upon cell damage in peripheral tissues induced by pathogens or allergens.

## Data Availability

The data presented in this study are available on request from the corresponding author.

## Supplementary Materials

The following supporting information can be downloaded at: https://www.mdpi.com/article/10.3390/cells12232696/s1, Figure S1: ATP and IL-33 synergistically increase the cytokine production in MCs. Figure S2: The SCF-, ATP- and IL-33-induced cytokine responses in MCs. Figure S3: The used inhibitors did not induce cell death.
