# Supplementary material for: ATP/IL-33-Co-Sensing by Mast Cells (MCs) Requires Activated c-Kit to Ensure Effective Cytokine Responses
Source: Cells. 2023 Nov 24;12(23):2696. doi: 10.3390/cells12232696 (PMC10705958; doi:10.3390/cells12232696)
Supplement: Supplementary file 1 [file cells-12-02696-s001.zip › cells-2665680-supplementary.pdf]

Figure S1

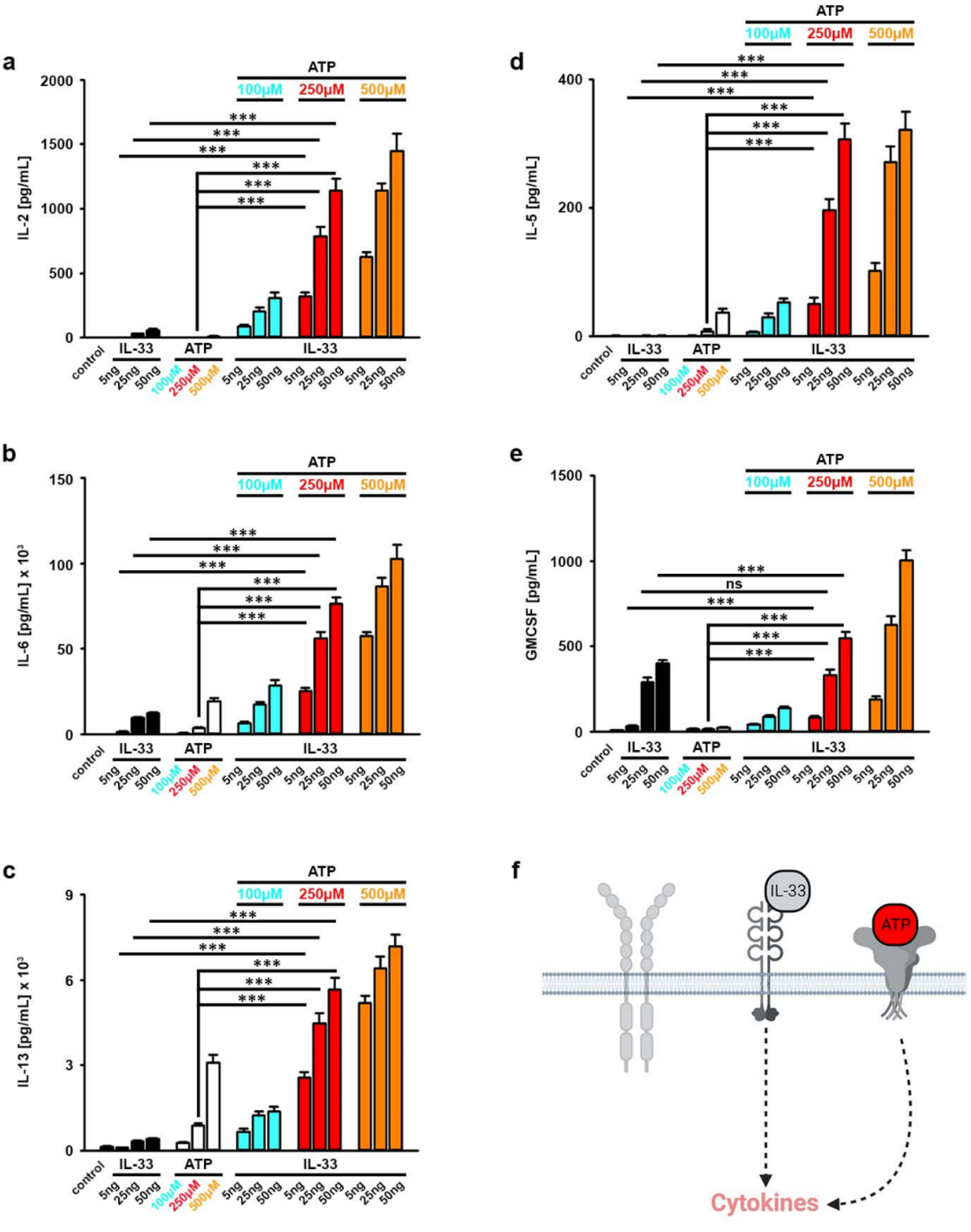

Supplementary Figure 1. ATP and IL-33 synergistically increase the cytokine production in MCs. (a-e) BMMCs cells were either single stimulated with ATP (as indicated) or IL-33 (as indicated) or both in combination. After 24h, supernatants were collected and analyzed for IL-2 **(a)**, IL-6 **(b)**, IL-13 **(c)**, IL-5 **(d)** or GM-CSF **(e)**. **(f)** Shown is the cartoon of the co-stimulation of BMMCs with ATP and IL-33 (created with biorender). Shown is the SEM of n=6 biological replicates for all cytokines. Shown is the SEM of n=6 biological replicates for IL-2, IL-6 and IL-13 and n=3 biological replicates for IL-5 and GM-CSF.

Figure S2

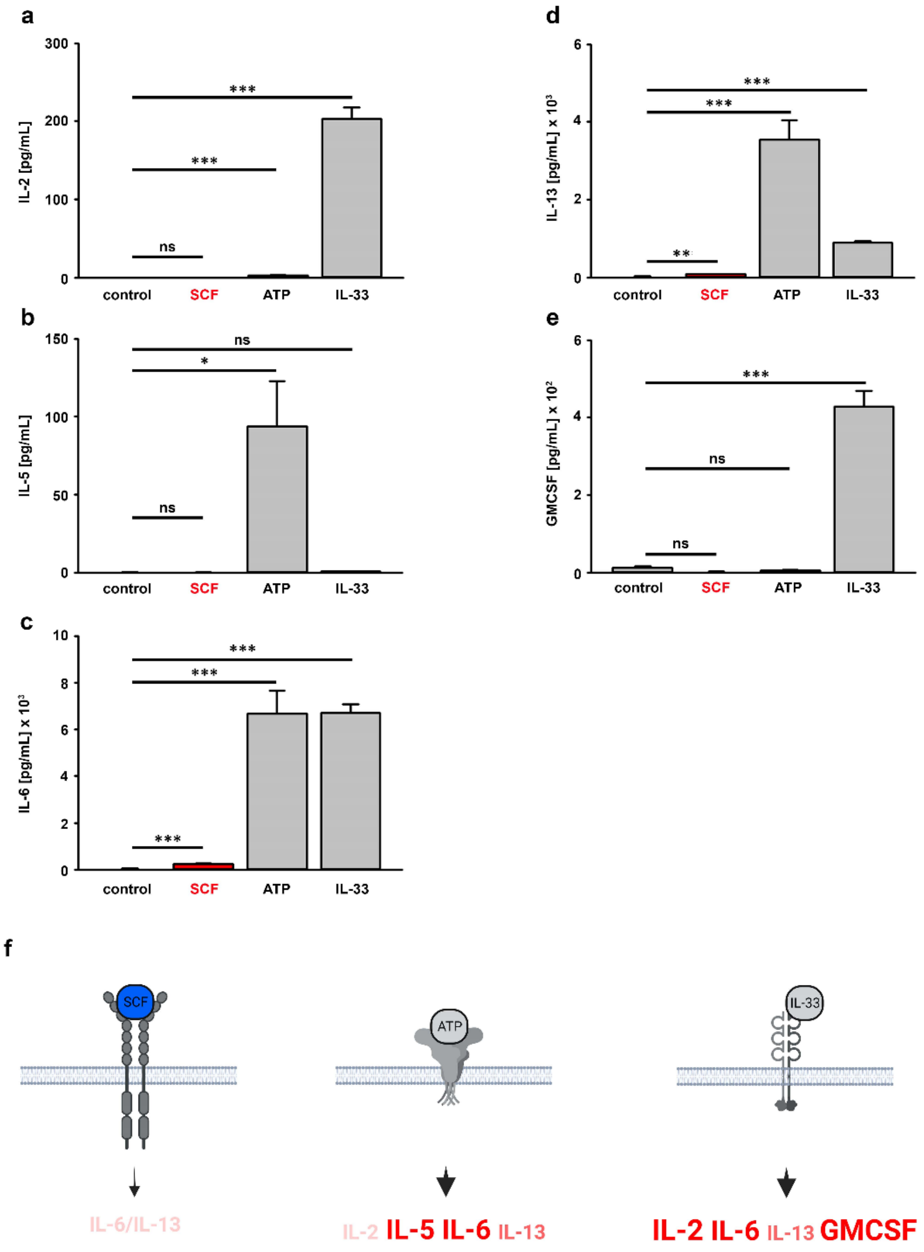

Supplementary Figure 2. The SCF-, ATP- and IL-33-induced responses in MCs. (a-e) BMMCs cells were single stimulated with SCF (50ng/ml), ATP (250µM) or IL-33 (25ng/ml). After 24h, supernatants were collected and analyzed for IL-2 **(a)**, IL-5 **(b)**, IL-6 **(c)**, IL-13 **(d)** or GM-CSF **(e)**. **(f)** Shown are the cartoons of the single stimulations of BMMCs with SCF, ATP or IL-33 and the resulting cytokine productions (created with biorender). **(a-e)** Shown is the SEM of n=3 biological replicates for all cytokines.

Figure S3

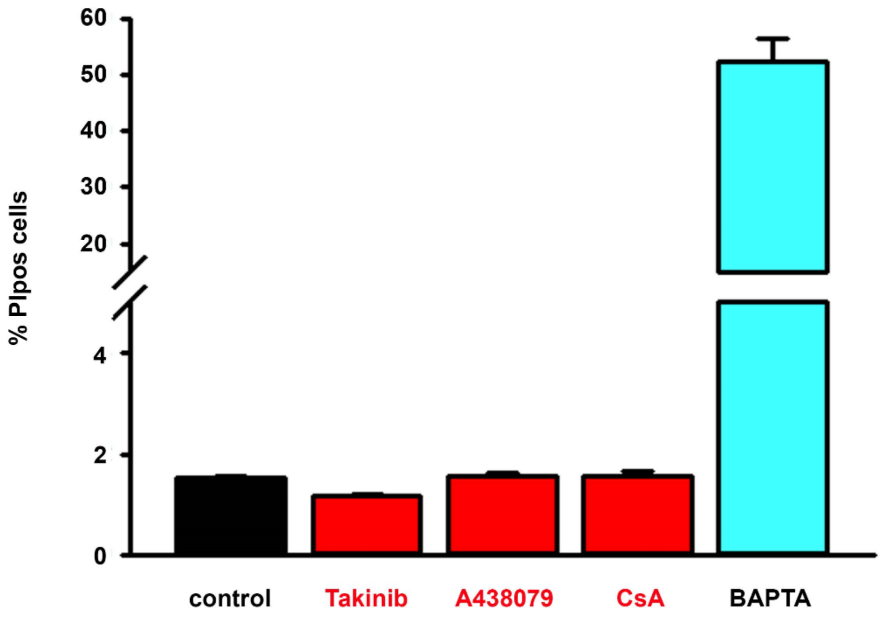

Supplementary Figure 3. The used inhibitors did not induce cell death. BMMCs were treated with Takinib 10µM, A438079 (50µM), CsA (2µg/ml) or with BAPTA (50µM) (as a positive control) which induces cell death in such high concentrations. After 24h cells were harvested, treated with propidium iodide, and analyzed by flow cytometry. Shown is the SEM of n=3 biological replicates.
